# Insertion of Iron Decorated Organic–Inorganic Cage-Like Polyhedral Oligomeric Silsesquioxanes between Clay Platelets by Langmuir Schaefer Deposition

**DOI:** 10.3390/ma13010216

**Published:** 2020-01-04

**Authors:** Jiquan Wu, Georgia Potsi, Regis Y. N. Gengler, Dimitrios Gournis, Petra Rudolf

**Affiliations:** 1 Zernike Institute for Advanced Materials, University of Groningen, Nijenborgh 4, 9747 AG Groningen, The Netherlands; j.q.wu689@gmail.com (J.W.); gepotsi@gmail.com (G.P.); ryn.gengler@gmail.com (R.Y.N.G.); 2 Department of Materials Science & Engineering, University of Ioannina, GR-45110 Ioannina, Greece; dgourni@uoi.gr

**Keywords:** Langmuir–Schaefer, polyhedral oligomeric silsesquioxanes, layered thin films, montmorillonite films

## Abstract

Tuning the architecture of multilayer nanostructures by exploiting the properties of their constituents is a versatile way to develop multifunctional films. Herein, we report a bottom-up approach for the fabrication of highly ordered hybrid films consisting of dimethyldioctadecylammonium (DODA), iron decorated polyhedral oligomeric silsesquioxanes (POSS), and montmorillonite clay platelets. Clay platelets provided the template where Fe/POSS moieties were grafted by the use of the surfactant. Driven by the iron ions present, DODA adopted a staggered arrangement, which is essential to realize the controllable layer-by-layer growth of the film. The elemental composition of the film was studied by X-ray photoelectron spectroscopy and X-ray reflectivity confirmed the existence of smooth interfaces between the different layers.

## 1. Introduction

Self-assembly provides the opportunity to create multifunctional hybrid materials from organic and inorganic building blocks [1]. Particularly self-assembly at surfaces can be exploited to build supramolecular architectures suitable for applications such as medical devices and catalyst supports, for many of which ordered thin films are required [2,3,4,5,6,7,8]. Langmuir–Schaefer (LS) deposition is a versatile room temperature growth method for thin films on rigid or flexible substrates; it also allows for an excellent control down to the molecular level by simple variations in the external parameters during deposition [9,10,11,12,13]. Our focus here is to synthesize new organic-inorganic hybrid materials by insertion of metal-decorated (Fe) polyhedral oligomeric silsesquioxane (POSS) between clay platelets through a modified LS method that alternates transfer of the Langmuir film and self-assembly.

In recent years, POSS have attracted significant attention because they can be used as templates for fabricating nanostructured materials such as star polymers [14,15,16], catalysts [17,18], and dendrimers [19]. In addition, POSS can be part of “self-healing” high temperature (nano)composites used in coatings [20,21]. POSS are made of a core-shell three-dimensional (3D) cage-like structure that can be fabricated through hydrolytic condensation reactions of organosilicon monomers RSiOH_3_ [22,23]. If properly functioned, POSS can be bonded with metal ions to form metal substituted silsesquioxanes [24,25].

Montmorillonite clay is a layered mineral, which can be intercalated, swell and serve as host for ion exchange. It has therefore been considered for application in various fields such as catalysis [26,27], synthesis templates [28,29], and building blocks for composite materials [30,31,32,33]. The structure of montmorillonite clay consists of an octahedral alumina sheet sandwiched between two tetrahedral silica sheets. It is negatively charged since part of the Al^3+^ ions in octahedral sites are substituted by Mg^2+^ and part of the Si^4+^ ions in tetrahedral sites are replaced by Al^3+^ [34]. These negatively charged platelets have the tendency to adsorb positive charged species (and even neutral molecules) on their surface and in the interlayer space between platelets [34].

Hybrid materials combining POSS and clay platelets have been synthesized for environmental remediation purposes. Deligiannakis et al. [35] intercalated amino-functionalized POSS in clays to produce a nanoporous adsorbent for heavy metals from aqueous solutions. In the same context Huh et al. [25] studied iron-modified POSS as adsorbent of cesium ions, methylene blue, and chrysoidine. Moreover, clay-POSS hybrids have been studied as catalysts [24] and novel additives in polymeric systems for the fabrication of nanocomposites since they improve thermal stability and thermal retardancy [36,37].

The challenge addressed in this work was to produce POSS-montmorillonite hybrids in thin film form, a step forward in making these hybrids available for a wider range of applications such as coatings. Differently from the bulk synthesis of POSS-intercalated clay [35], the LS technique allows to produce multilayer hybrids with outstanding control over the thickness as well as the structure [34,38,39,40]. Considering these advantages, in this work, we aim at fabricating nanostructured thin films where iron decorated POSS is sandwiched between organo-modified clay platelets. Iron was chosen as the metal ion grafted to POSS because of its low toxicity and high abundance that render it an excellent candidate for industrial applications [24]. The proposed experimental approach provides an alternative for hybrid synthesis that avoids aggregation phenomena; the latter are undesirable in applications such as anti-reflection coatings with high and low refractive index multilayers [41] or photofunctional films [42].

## 2. Materials and Methods

### 2.1. Materials

Kunipia F (KUN), a natural dioctahedral montmorillonite obtained from Kunimine Industries Co. (Tokyo, Japan), was used in this work. Its structural formula is Ca_0.11_Na_0.891_(Si_7.63_Al_0.37_)(Al_3.053_Mg_0.65_Fe_0.245_Ti_0.015_)O_20_(OH)_4_ and its cation exchange capacity (CEC) 1.18 meq/g. Dimethyldioctadecylammonium (DODA, Sigma-Aldrich Chemie N.V., Zwijndrecht, The Netherlands) and FeCl_2_ 4H_2_O (99%, Sigma-Aldrich Chemie N.V., Zwijndrecht, The Netherlands) were used as received. Pieces of 10 × 10 mm^2^ of a 280-nm thick silicon oxide/silicon wafer (purchased from Silicon Quest International, San Jose, CA, USA) were cleaned by sonication in acetone and in iso-propan-2-ol ((CH_3_)_2_CHOH) for 5 min; then the wafer pieces were sonicated in Milli-Q water for 5 min and rinsed in Milli-Q water before being dried by spinning and blowing with N_2_ (99.995%) gas. Before being used as substrates in our deposition experiments these substrates were made hydrophobic as described in [43] by modifying them with octadecyltrichlorosilane (Sigma-Aldrich Chemie N.V., Zwijndrecht, Netherlands).

### 2.2. Synthesis of Polyhedral Oligomeric Silsesquioxanes (POSS)

To synthesize POSS, the controlled hydrolysis of 3-(2-aminoethylamino)-propyltrimethoxysilane in an ethanol/water 14:1 *v*/*v* solution was performed. Simultaneously 30 mL of an aqueous FeCl_2.4_H_2_O (0.1 mol/L) solution was prepared and added to 20 mL of the above solution; incubation of the mixture for 24 h engendered the formation of iron decorated cage-like species [24,44,45].

### 2.3. Preparation of Films by a Modified Langmuir–Schaefer Approach

**Scheme 1:**
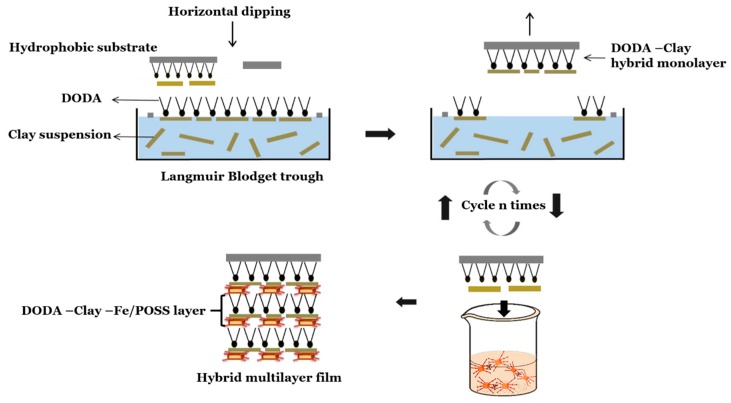
Preparation procedure and suggested model of multilayer dimethyldioctadecylammonium (DODA)-clay-Fe/silsesquioxanes (POSS) film.

For Langmuir–Schaefer (LS) deposition, a dilute (10 ppm) suspension of negatively charged clay nanosheets was used as subphase (employing MilliQ water, resistivity > 18 MΩ cm) into a Nima Technology thermostated 612D Langmuir Blodgett (LB) trough kept at 23 ± 0.5 °C; then DODA, a cationic surfactant with a positively charged terminal group and a hydrophobic tail, dissolved in chloroform-ethanol (9:1 *v*/*v*), was deposited at the surface of the clay suspension (air water interface) with the help of a microsyringe. The positively charged DODA terminal group attracts the negatively charged clay nanosheets to the surface through electrostatic interactions. The surfactant molecules with the attached clay platelets behave like a 2D gas, which can be compressed with the help of the movable barriers to form a close-packed Langmuir film in which the hydrophobic alkyl chains point away from the water surface [46,47,48,49]. In our experiments we allowed for a 30 min waiting time for chloroform to evaporate before starting compression at a rate of 25 cm^2^/min. Gengler et al. [34] studied the Π-a isotherms of DODA monolayers on clay dispersions with concentrations ranging from 5 to 500 ppm. Based on those results we chose a clay suspension with the optimized concentration of 10 ppm in order to avoid the aggregation of DODA and clay nanosheets during deposition process. For the thin films fabrication we chose the target surface pressure 15 mN/m that was maintained constant during the whole deposition process. The multilayer organic–inorganic hybrid LS film was prepared by alternating LS and self-assembly deposition. In particular, a DODA-clay layer is deposited by dipping the substrate into the LB trough (downstroke speed 4 mm min^−1^, upstroke speed 2 mm min^−1^) and then a layer of iron-decorated POSS is grafted onto the clay surface through self-assembly by lowering the sample into a 50 mL solution of Fe/POSS (30 mL of 0.1 M FeCl_2_ mixed with 20 mL of 0.45 M POSS) as illustrated in Scheme 1. This procedure was repeated several times for the fabrication of hybrid multilayered nanostructures. After each deposition step, the substrate was dipped into MilliQ water and blown dry with nitrogen to prevent any contamination of the Langmuir–Blodgett trough and/or the Fe/POSS solution.

### 2.4. Characterization Methods

X-ray photoelectron spectroscopy (XPS) was performed with a Surface Science SSX-100 ESCA instrument, equipped with a monochromatic Al Kα X-ray source (hν = 1486.6 eV) and operating at a base pressure of ~5 × 10^−10^ mbar. The energy resolution was set to 1.26 eV (or 1.67 eV for a broad survey scan) and the electron take-off angle was 37° with respect to the surface normal. The XPS spectra were analyzed using the least-squares curve fitting program Winspec, developed at the LISE laboratory, University of Namur, Belgium. Binding energies deduced from curve fitting are reported to a precision of ±0.1 eV and referenced to the C1*s* photoemission peak at 284.5 eV [50]. All measurements were carried out on freshly prepared samples, three different spots were measured on each sample to check for homogeneity of the film.

X-ray reflectivity (XRR) experiments were carried out on 34 layer-thick DODA-clay-Fe/POSS hybrid films. Data were collected under ambient conditions with a Philips PANanalytical X’Pert MRD diffractometer, equipped with a Cu Kα (λ = 1.5418 Å) X-ray source (operated at 40 kV, 40 mA); 2θ scans were performed from 0.6° to 15° (0.02° step, counting time of 15 s per step).

## 3. Results and Discussion

**Figure 1:**
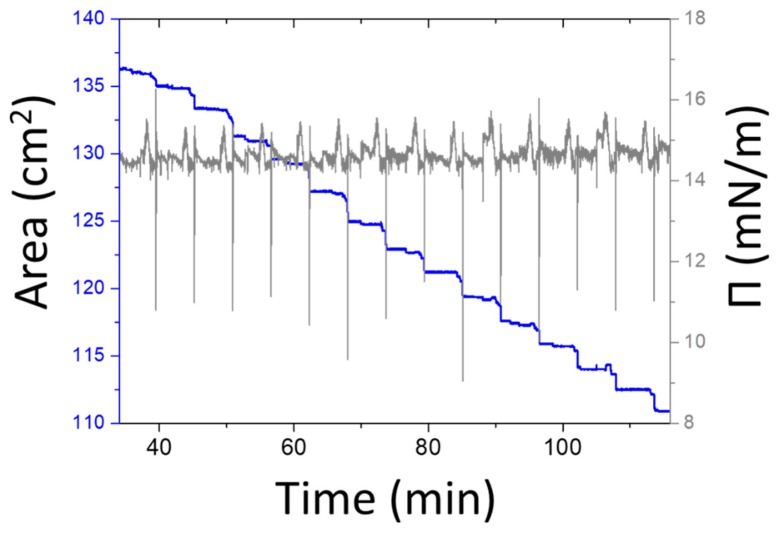
Langmuir Schaefer deposition of a DODA-clay-Fe/POSS hybrid film. The blue curve represents the trough area covered by the Langmuir film; the grey curve denotes the change of the surface pressure throughout the whole deposition.

To gain information on the film quality yielded by the modified Langmuir–Schaefer method, we recorded the time dependence of the surface pressure and the total area covered by the Langmuir film during the deposition process illustrated in Scheme 1. The results are shown in Figure 1 and testify to the successful transfer of the DODA-clay layer. When the substrate is horizontally dipped into the trough, the Langmuir film area reduces owing to the transfer of the DODA-clay layer from the air–water interface to the substrate [12]; this process generates a sharp step in the area versus time curve (blue line) and a sharp downward peak on the curve of pressure versus time (black line) in Figure 1. The step height indicates the area that is transferred to the substrate. The transfer ratio is 1 if the decrease in area equals the substrate area. Given that the substrate area was ~2.5 cm^2^, we calculated that the transfer ratio was ~0.9, verifying the successful transfer of the hybrid Langmuir film (DODA-clay) at each deposition.

### 3.1. XRR Pattern of a DODA-Clay-Fe/POSS Hybrid Film

**Figure 2:**
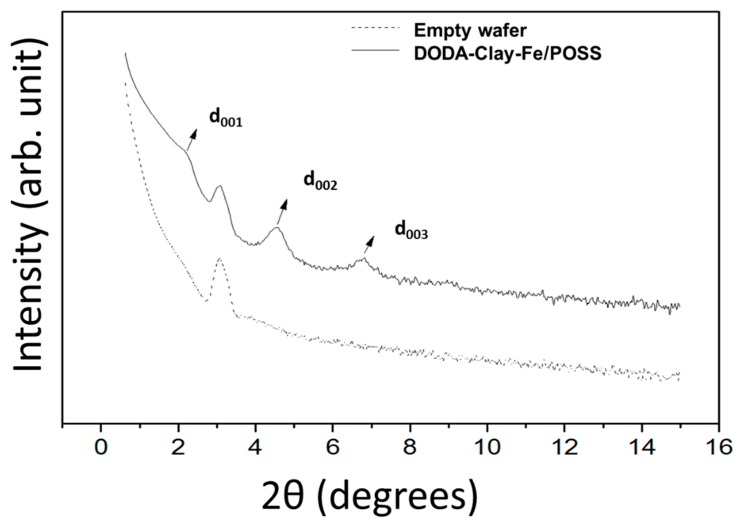
X-ray reflectivity patterns of 34-layer thick DODA-clay-Fe/POSS hybrid film deposited following the preparation route in Scheme 1.

X-ray reflectivity (XRR) studies inform on the hybrid film structure and can hence attest the quality of films resulting from our deposition protocol. Figure 2 shows the specular X-ray reflectivity of a 34-layer thick DODA-clay-Fe/POSS hybrid film. Bragg peaks were observed at 2θ = 2.24° ± 0.05°, 4.57 ± 0.05°, and 6.80 ± 0.05°, i.e., at equal increments characteristic of (00l) reflections of layered materials. The smallest spacing *d* of the periodic unit perpendicular to the film surface was deduced from the diffraction peak positions via the Bragg formula. The *d* value found for the DODA-clay-Fe/POSS film was 39.5 ± 0.5 Å. An extra peak appeared between d_001_ and d_002_ as shown in Figure 2; by collecting data also from an empty wafer substrate (lower curve in Figure 2) we confirmed that this peak is an artifact introduced by the X-ray diffractometer.

**Scheme 2:**
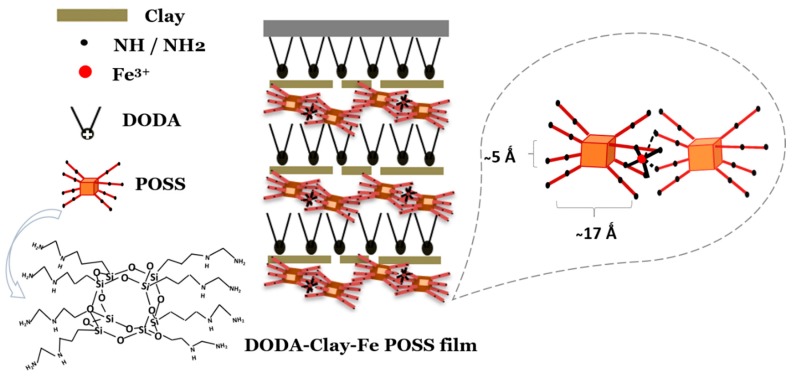
Model of the arrangement of the POSS units in the DODA-clay-Fe/POSS films, which explains the X-ray reflectivity results.

The thickness of the DODA monolayer can vary depending on the tilting angle that the molecules adopt in the film [51,52] and can reach 25 Å. The thickness of a clay platelet is 9.6 Å [53]. As to the dimensions of POSS, when the flexible side chains take a horizontal orientation, the POSS layer is at least 5 Å thick, [35,54] while it accounts for a thickness of 17–17.6 Å [54] when the side chains take a vertical orientation as indicated in Scheme 2. We will discuss the arrangement of POSS between clay platelets when we examine the XPS data below. Given that we found a *d* value of about 40 Å, it is reasonable to assume that the smallest repeat unit consists of one DODA layer, one clay platelet, and one layer of Fe-decorated POSS. If we consider the deposition procedure sketched in Scheme 1, when the substrate is lifted from the POSS solution, the DODA-clay-Fe/POSS layer formed is positively charged, while the DODA monolayer at the subphase surface is terminated by hydrophobic alkyl chains. When the substrate is lowered into the LB trough again, the positively charged surface layer interacts with the DODA-clay Langmuir film and the latter is transferred, thus giving rise to an X-type structure in the film. A similar mechanism of forming multilayer hybrid structures was described by Umemura et al. [49].

### 3.2. Probing the Surface of DODA-Clay-Fe/POSS by XPS

**Figure 3:**
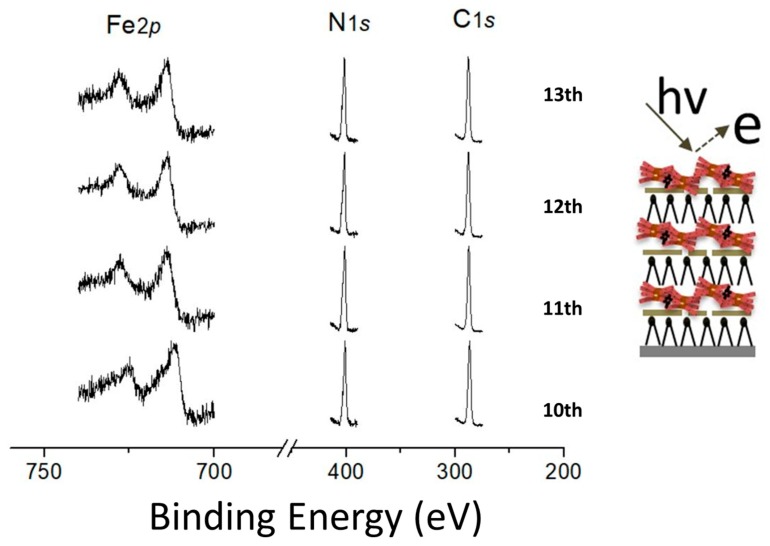
X-ray photoemission spectra of the Fe2*p*, N1*s*, and C1*s* (used as reference) core level regions collected from 10- to 13-layer-thick DODA-clay-Fe/POSS hybrid films.

To verify whether the multilayer really adopts an X-type structure as proposed above, we performed X-ray photoelectron spectroscopy (XPS) measurements on films prepared following the preparation route illustrated in Scheme 1. XPS is an ideal tool to identify the surface elemental composition of solids. Therefore, we used this technique to probe the topmost surface of DODA-clay-Fe/POSS hybrid films. Figure 3 shows the C1*s* (used as reference), N1*s*, and Fe2*p* core level photoemission signals collected from DODA-clay-Fe/POSS hybrid films of different thickness. It can be clearly seen that the intensities of Fe and N peaks are the same for samples with an even or an odd number of layers. This observation confirms the X-type structure. An alternative would have been a Y-type structure, achieved by a “flip over” of DODA layers during the deposition [34]. If the “flip over” of DODA layers had taken place, the intensity of peaks attributed to elements belonging to moieties grafted onto the clay platelets, in this case the N1*s* peak due to POSS, should be observed to decrease and increase for alternate layer thicknesses [34].

**Figure 4:**
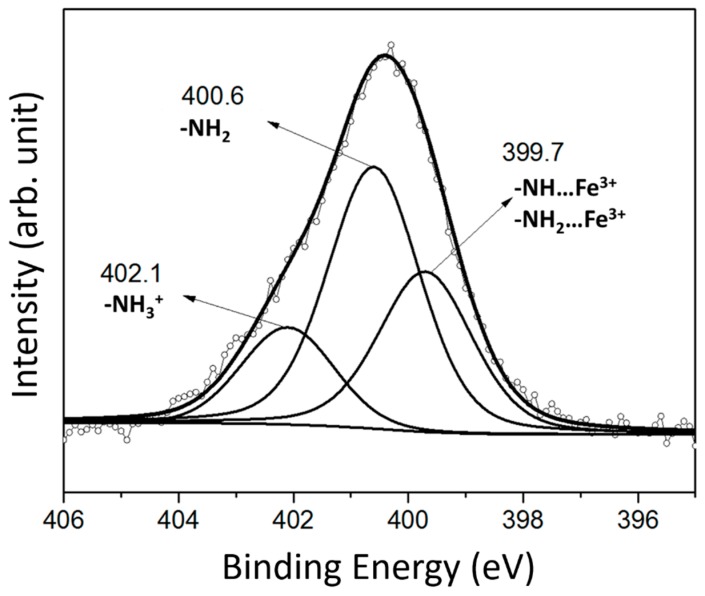
X-ray photoemission spectrum of the N1*s* core level region of a 13-layer-thick DODA-clay-Fe/POSS hybrid film.

However, an X-type film structure does not yet explain the spacing of about 40 Å. To justify why the POSS units are bound to one another in a staggered fashion as sketched in Scheme 2 which leads to a thickness of the POSS layer >5 Å, we have to inspect the N1*s* core level photoemission spectrum in detail. As evident in Figure 4, the N1*s* line of the DODA-clay-Fe/POSS hybrid film consists of three components observed at 399.7, 400.6, and 402.1 eV. The peak at a binding energy of 400.6 eV, accounting for 50.8% of the total N1*s* spectral intensity, stems from primary amine groups (-NH_2_) and the component at a binding energy of 402.1 eV, which contributes for 17.5% to the total N1*s* spectral intensity, is attributed to protonated amine end groups (NH_3_^+^) that are positively charged and connected to the negatively charged clay sheets [55]. The third peak at 399.7 eV, which makes up 31.7% of the total N1*s* spectral intensity, results from nitrogen groups (R-NH-R and R-NH_2_) coordinated with Fe^3+^ ions [56]. This last species drives the geometry of the DODA-clay-Fe/POSS film; the coordination of iron (six ligands available) brings about the linking of a second silsesquioxane and the creation of a staggered layer during film deposition, as sketched in Scheme 2.

## 4. Conclusions

We demonstrated the successful insertion of iron decorated organic-inorganic polyhedral oligomeric silsesquioxanes (POSS) between clay platelets, to form a well-ordered hybrid film when deposited in a layer-by-layer fashion. Hybrid films were fabricated by alternating Langmuir–Schaefer deposition and self-assembly steps, with precise control of the structure built up during the growth. This approach can also serve for different hybrid films where the molecular organization within the system can be manipulated by choosing different organic or inorganic species. Moreover, this alternative synthesis technique for hybrids avoids undesirable aggregation phenomena that are often encountered in bulk synthesis [57].
